# Quantifying indirect and direct vaccination effects arising in the SIR model

**DOI:** 10.1098/rsif.2024.0299

**Published:** 2024-09-18

**Authors:** Lixin Lin, Homayoun Hamedmoghadam, Robert Shorten, Lewi Stone

**Affiliations:** ^1^ Mathematical Sciences, School of Science, RMIT University, Melbourne, Australia; ^2^ Dyson School of Design Engineering, Imperial College London, London, UK; ^3^ Biomathematics Unit, School of Zoology, Faculty of Life Sciences, Tel Aviv University, Tel Aviv, Israel

**Keywords:** SIR model, epidemics, indirect vaccination effects, shielding, imperfect vaccination, herd immunity

## Abstract

Vaccination campaigns have both direct and indirect effects that act to control an infectious disease as it spreads through a population. Indirect effects arise when vaccinated individuals block disease transmission in any infection chain they are part of, and this in turn can benefit both vaccinated and unvaccinated individuals. Indirect effects are difficult to quantify in practice but, in this article, working with the susceptible–infected–recovered (SIR) model, they are analytically calculated in important cases, through pivoting on the final size formula for epidemics. Their relationship to herd immunity is also clarified. The analysis allows us to identify the important distinction between quantifying the indirect effects of vaccination at the ‘population level’ versus the ‘per capita’ level, which often results in radically different conclusions. As an example, our analysis unpacks why the population-level indirect effect can appear significantly larger than its per capita analogue. In addition, we consider a recently proposed epidemiological non-pharmaceutical intervention (by the means of recovered individuals) used over the COVID-19 pandemic, referred to as ‘shielding’, and study its impact on our mathematical analysis. The shielding scheme is extended to take advantage of vaccination including imperfect vaccination.

## 1. Introduction

Vaccination campaigns have both direct and indirect effects on the transmission of an infectious disease as it spreads through a population [1–4]. Direct vaccination effects refer to the reduction in the risk of infection due to the protection provided to individuals by the vaccine dose. Indirect vaccination effects refer to the protection provided to an individual by nearby vaccinated neighbours that indirectly act to block chains of incoming infections, or just reduce infection possibilities, thereby ‘shielding’ the individual [3–5]. The indirect effect of vaccination, via such shielding, makes it more difficult for a disease to spread in a population and hence also affects conditions that give rise to herd immunity [6]. Indirect vaccination effects are difficult to quantify in practice, but clearly understanding the role and magnitude of indirect effects is critical for the design of vaccination programmes [2–4]. A deeper theoretical analysis is still lacking and there is a need to better formulate conditions that predict when the impact of indirect effects will be significant and when they will be minor [2–4,7]. Here, we make use of simple mathematical models to achieve this goal, and in important cases succeed to give an exact characterization. In the process, we make clear the important distinction of quantifying indirect effects at the population level or as a ‘per-capita’ quantity, a distinction that is often neglected.

Haber [3] gives a simple elegant example that shows how indirect effects matter and how their ‘shielding’ property can be taken advantage of. ‘Consider a population that consists of 10 000 individuals in 2000 households so that the average household size is 5. If 4000 vaccine doses are available, what is the best way to distribute the vaccine? Perhaps, the simplest way is to select 800 households and vaccinate everyone in these households. However, an alternative plan, is to vaccinate two persons in each of the 2000 households. The second plan is more effective, because it utilizes the indirect effects of shielding to protect the unvaccinated household members.’ In fact, indirect effects endow additional protection to all households and individuals in the second plan, in contrast to the first plan that leaves 1200 households with no vaccine protection.

The most striking example of indirect effects relates to herd immunity, where the theory predicts that there is no need to vaccinate more than a critical proportion of a population to completely protect the whole population from a disease [6,8,9]. The herd immunity threshold can be predicted, for example, for the simple classical susceptible–infected–recovered (SIR) epidemic model that will be described shortly. At the herd immunity threshold, if a proportion *v* of the population is ‘directly’ vaccinated, then the proportion (1 − *v*) of the population does not require vaccination since it will be protected indirectly by herd immunity effects [6,8]. The division of the population in this ratio *v*/(1 − *v*) (i.e. direct : indirect) proves to be important in what follows.

As mentioned, quantifying the direct and indirect effects is of great value in the evaluation of vaccination campaigns [1–4,10]. But it is a complicated procedure. In a number of studies, the indirect vaccination effect has been estimated to be unusually large in magnitude. Scutt *et al*. [11] show that in some cases the indirect vaccination effect can be more than 400% of the direct vaccination effect. Eichner *et al*. [4] discuss an example in Canada, where ‘vaccination of 83% of children (≤15 years) reduced influenza infection incidence in unvaccinated individuals by 61%’. Similarly, in modelling COVID−19, Gavish *et al*. [12] find that among the cases reduced as a result of the booster campaign, approximately 54% were reduced because of direct protection, whereas the remainder were reduced by indirect protection. Gallagher *et al*. [13] highlight the critical importance of considering the indirect vaccination effect when evaluating SARS-CoV-2 vaccine candidates. Through modelling, they emphasize the importance of not automatically selecting a vaccine based solely on the largest direct effect. Weidemann *et al*. [7] point out that there are exceptions and that some studies report that the impact of indirect effects can be low (e.g. as in the school vaccination programmes on the US county level [14,15]).

Here, the extent of indirect effects and the factors that enhance them are explored by means of mathematical analysis. In contrast to other analyses [4,11], we use the final size formula [16] of the epidemic, which allows us to draw analytical conclusions from the mathematical models without relying on numerical simulations. We also extend the original shielding scheme [5] to allow for vaccination and apply our analysis of the vaccination effects to the extended shielding model. Initially, we assume perfect vaccines (with 100% efficacy) and then extend the analysis to imperfect vaccines (both ‘leaky’ and ‘all-or-nothing’ vaccines).

## 2. SIR epidemics with vaccination

The standard SIR model assumes that at any time t , each individual in a population can only belong to one of three classes: susceptible**,** infected or recovered. Taking the proportions of individuals as S , I or R , then clearly S(t)+I(t)+R(t)=1 . In a randomly mixing population, new infections are generated when infected individuals come into contact with susceptible individuals at a rate proportional to the product S⋅I , which leads to the following well-known SIR equations:


(2.1) $$\begin{array}{ll} \frac{dS}{dt}\; & =\;-\;\beta SI,\\ \frac{dI}{dt}\; & =\;\beta SI\;-\;\gamma I,\\ \frac{dR}{dt}\; & =\;\gamma I. \end{array}$$

In this scheme, over time individuals move from the S class to the I class, and finally end up in the recovered R class, i.e. S→I→R . Here β is the transmission rate ( S→I ) between individuals, while γ is the recovery rate ( I→R ). The basic reproduction number is defined as $R_{0}\;=\;\beta /\gamma$ . Initial conditions are such that almost all members of the population are susceptible, and the number of initially infected individuals is infinitesimally small, so that we may approximate S(0)=1 , I(0)=R(0)=0 . According to standard theory, for these initial conditions, if a single infected individual enters the system, an epidemic will occur only if $R_{0}\;>\;1$ , since that ensures dI/dt > 0 at t=0.

A simple model of vaccination can be explored by changing the initial conditions. Suppose that initially a proportion v of a fully susceptible population has been vaccinated and has thus become fully protected from infection. The population’s vaccination coverage is said to be v , and we set


(2.2) $$\begin{array}{c} S\left(0\right)\;=\;1\;-\;v,\;\;\;I\left(0\right)\;=\;0,\;\;\;R\left(0\right)\;=\;v. \end{array}$$

In this case, standard theory shows that an epidemic can only occur if the effective reproduction number $R_{e}\;=\;R_{0}\;S\left(0\right)\;>\;1$ , which is equivalent to having a vaccination level $v\;<\;v_{h}$ , where $v_{h}$ is the vaccination coverage at the herd immunity threshold and is defined as


(2.3) $$v_{h}\;=\;1\;-\;\frac{1}{R_{0}}.$$

Under this condition, the epidemic’s final size $Z\left(v\right)$ (or equivalently attack rate) is the proportion of the population infected over the epidemic and is measured with the solution of the following equation [16]:


(2.4) $$\begin{array}{c} Z\left(v\right)\;=\;\left(1\;-\;v\right)\left(1\;-\;e^{-R_{0}Z\left(v\right)}\right). \end{array}$$

**Figure 1.**
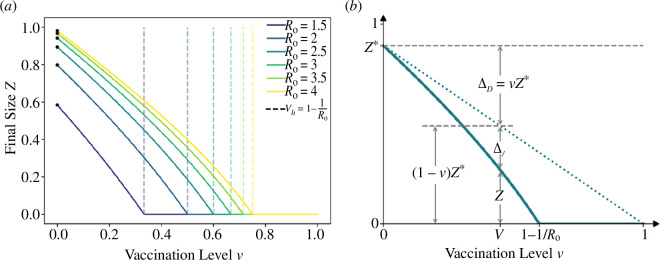
(*a*) The relationship between the final size of the epidemic and the proportion v of individuals vaccinated for $R_{0}\;=\;1.5,\;2,\;\;2.5,\;\;3,\;\;3.5,\;\;4$ , calculated by solving equation (2.4). Note that the herd immunity threshold, e.g. for $R_{0}\;=\;2.5$ is at v=0.6 . The marked black points identify the final size of the epidemic when v=0 , i.e. $Z^{*}$ for different values of $R_{0}$ . (*b*) The relationship between the proportion of individuals vaccinated, v , and the infections averted by direct vaccination effect $\Delta_{D}\;=\;vZ^{*}$ , and indirect vaccination effect $\Delta_{I}\;=\;\left(1\;-\;v\right)Z^{*}\;-\;Z$ . See equations (2.8) and (2.9).

The solid lines in figure 1*a* show the final size Z(v) as a function of vaccination v , repeated for different values of the reproduction number $R_{0}$ . In the absence of vaccination ( v=0 ), the final size $Z^{*}\;=\;Z\left(0\right)$ is given by the solution of


(2.5) $$\begin{array}{c} Z^{*}\;=\;1\;-\;e^{-R_{0}Z^{*}}. \end{array}$$

The final size in the absence of vaccination ( $Z^{*}$ ) is marked by black points in figure 1*a*, as a reference indicating the case where v=0 . Thus, for a high reproduction number (e.g. $R_{0}\;=\;4$ , yellow curve) and no vaccination ( v=0 ), the final size is $Z^{*}\;=\;0.99$ and the epidemic would infect 99% of the population. The final size decreases with increasing v , and for any $R_{0}$ is zero at the herd immunity threshold $v\;=\;v_{h}\;=\;1\;-\;1/R_{0}$ . Given $R_{0}\;=\;4$ , at the herd immunity threshold ( v=0.75 ), an epidemic is not possible despite the fact that 25% of the population remain unvaccinated and thus susceptible. In this situation, the vaccination is protecting the unvaccinated population indirectly.

### 2.1. Calculating direct and indirect effects of vaccination

For any level of vaccination coverage v , it is possible to determine the total proportion of the population protected by the vaccine or equivalently the proportion ${∆}_{T}$ of infections that has been averted, as compared with the situation had there been no vaccination. Most methods use simulation or approximation techniques to achieve this [4,11,12]. Here, however, we find a solution based on the final size equation for the SIR model. The proportion ${∆}_{T}$ is simply the difference between the final size with no vaccination $Z^{*}$ and the final size with vaccination Z=Z(v) , namely,


(2.6) $$\begin{array}{c} \Delta_{T}\;=\;Z^{*}\;-\;Z. \end{array}$$

The proportion of total infections averted, ${∆}_{T}$ , as a function of v is visualized by the gap between the solid line for Z and the dashed line for $Z^{*}$ in figure 1*b*. It is important to note that ${∆}_{T}$ has two components:

— the direct vaccination effect ( ${∆}_{D}$ ), which is the proportion of infections averted among the vaccinated individuals who gained protection directly from the vaccine and
— and the indirect vaccination effect ( ${∆}_{I}$ ), which is the proportion of infections averted among the unvaccinated individuals due to population-level immunity.

That is


(2.7) $$\begin{array}{c} \Delta_{T}\;=\;\Delta_{D}\;+\;\Delta_{I}. \end{array}$$

It is not straightforward to disentangle the two components ${∆}_{D}$ and ${∆}_{I}$ . In the case of the SIR model, since there is no vaccinated compartment, it is difficult to track the fate of vaccinees and thus obtain the direct effect ${∆}_{D}$ . Eichner *et al.* [4] describe a method that adds a vaccinated compartment and simulates the number of infections generated by vaccinees who received a completely ineffective vaccine. Tallying the total number of new infections generated from this subgroup gives the direct effect. Based on a similar concept, we show, using a final size formulation, that the proportion of infections directly averted ${∆}_{D}$ is given by


(2.8) $$\begin{array}{c} \Delta_{D}\;=\;v\cdot Z^{*}, \end{array}$$

where $Z^{*}\;=\;Z\left(0\right)$ , is the size of the epidemic in the absence of vaccination. Electronic supplementary material (note 1) provides a proof based on the continuous-time SIR model.

Equation (2.8) is an important result that is also to some degree intuitive. For example, if the epidemic infected all members of the population $\left(Z^{*}\;=\;1\right)$ , it is clear that the proportion of those infected who are vaccinated is $\Delta_{D}\;=\;vZ^{*}$ . The relationship in equation (2.8) was also noted by Scutt *et al*. [11] for a different discrete-time epidemic model.

From equations (2.6) to (2.8), the proportion of infections averted by indirect vaccination effect as a function of v is


(2.9) $$\Delta_{I}\;=\;\Delta_{T}-\Delta_{D}\;=\;Z^{*}-Z-v\;Z^{*}\;=\;\;\left(1\;-v\right)Z^{*}-Z\;\;\leq \;\left(1\;-v\right)Z^{*},$$

where again we use the notation Z=Z(v) . The indirect effect thus comprises all unvaccinated individuals that would have been infected in the absence of vaccination $\left(1\;-\;v\right)Z^{*}$ , but taking away the total number of infected individuals under a vaccination programme (see figure 1*b*).

Notice in figure 1*a* that the final size as a function of *v* may be roughly approximated by the linear relationship $Z\;=\;Z^{*}\;\left(1\;-\;v/\left(1\;-\;1/R_{0}\right)\right)$ , as long as $v\;\leq \;v_{h}$ . The approximation ensures that $Z\left(0\right)\;=\;Z^{*}$ , and at the herd immunity threshold $v_{h}\;=\;1\;-\;1/R_{0}$ , it gives $Z\left(v_{h}\right)\;=\;0.$ Substituting this linear expression into equation (2.9) results in


(2.10) $$\Delta_{I}≃Z^{*}-\;Z^{*}\;\left(1-\frac{v}{1-\frac{1}{R_{0}}}\right)\;-\;v\;Z^{*}\;=\;\frac{v\;Z^{*}}{R_{0}-1\;}.$$

The proportions of infections averted by direct ( $\Delta_{D}$ ) and indirect ( $\Delta_{I}$ ) vaccination effect are shown in figure 1*b*, found from solving equations (2.4)–(2.9). In figure 1*b*, the dotted green line shows the linear relationship between $\left(1\;-\;v\right)Z^{*}$ and v . Thus, the infections averted by the direct vaccination effect ${∆}_{D}$ is the gap between the dashed and the dotted lines, and the infections averted by the indirect vaccination effect ${∆}_{I}$ is the gap between the dotted and the solid green lines.

**Figure 2.**
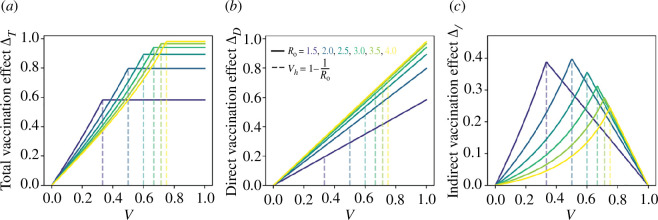
The proportion of infections averted by vaccination as a function of vaccination level v . (*a*) Total vaccination effect ${∆}_{T}$ ; (*b*) direct vaccination effect ${∆}_{D}$ ; (*c*) indirect vaccination effect ${∆}_{I}$ . This is repeated for $R_{0}\;=\;1.5,\;\;2.0,\;\;2.5,\;\;3.0,\;\;3.5,\;\;4.0$ . The dashed lines in each panel represent the ‘herd immunity’ vaccination coverage levels $v_{h}\;=\;1\;-\;1/R_{0}$ , with values from left to right corresponding to increasing $R_{0}$ values.

Figure 2 unpacks the impact of the vaccination effect as a function of v . The infections averted by total, direct and indirect vaccination effects, respectively, are plotted in figure 2 as a function of v , for $R_{0}$ = 1.5, 2.0, 2.5, 3.0, 3.5, 4.0. These results have been corroborated in electronic supplementary material (note 2) through simulations of an SIRV model.

There are several interesting features seen in figure 2. First, the total infections averted by vaccination ${∆}_{T}$ clearly increases with *v*, until the herd immunity threshold is reached at $v_{h}\;=\;\;1\;-\;1/R_{0}$ . Hence, the most effective campaign is the one with the highest vaccination levels despite the presence of prominent indirect effects at lower vaccination levels. Second, the proportion of infections averted by the direct vaccination effect ${∆}_{D}$ is linear in v . In contrast, the proportion of infections averted by the indirect vaccination effect ${∆}_{I}$ increases nonlinearly in v as long as $v<\;v_{h}\;=\;1-1/R_{0}$ . Beyond the herd immunity threshold ( $v\;>\;v_{h}$ ), the indirect effect decreases linearly until $\Delta_{I}\;=\;0$ is reached when v=1 . When v is increased beyond the herd immunity threshold $v_{h}$ , the epidemic has died out and there are zero infectives in the population. Since the effective reproduction number at t=0 is given by $R_{e}\left(0\right)\;=\;R_{0}\;S\left(0\right)\;=\;R_{0}\;\left(1-v\right)$ , we can see that the indirect vaccination effect reaches its maximum when $v\;=\;v_{h}\;=\;\;\left(1\;-\;1/R_{0}\right)$ , that is, when $R_{e}\left(0\right)\;=\;1$ .

### 2.2. Ratio of indirect to direct vaccination effects

According to the formulae developed so far, the ratio of indirect to direct vaccination effects is


(2.11) $$\frac{\Delta_{I}}{\Delta_{D}}\;=\;\frac{\left(1-v\right)Z^{*}-Z}{v\;Z^{*}}\;=\;\frac{1-v}{v}\;-\;\frac{Z}{v\;Z^{*}}.$$

**Figure 3.**
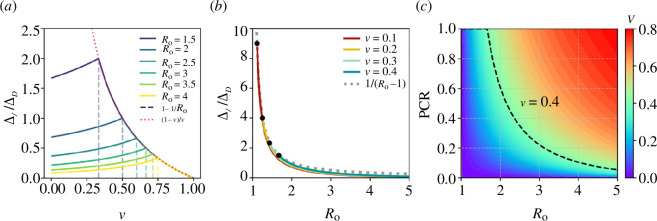
(*a*) The ratio of indirect to direct vaccination effects ${∆}_{I}/{∆}_{D}$ versus the vaccination level v , for different values of $R_{0}$ , using equation (2.11). The dashed lines (each associated with a curve of the same colour) indicate where herd immunity was reached for a particular value of $R_{0}$ . The dotted red curve shows (1−v)/v , which the ratio collapses to when $v\;\geq \;v_{h}$ for any value of $R_{0}$ (see equation (2.12)). (*b*) The ratio of indirect to direct vaccination effects ${∆}_{I}/{∆}_{D}$ as a function of $R_{0}$ for different values of v . The dashed grey line plots $1/\left(R_{0}\;-\;1\right)$ ; see equation (2.16). (*c*) PCR as a function of $R_{0}$ for different vaccination levels v between 0 and 0.8. The dashed black curve shows the trend in PCR versus increasing $R_{0}$ for v=0.4 .

In figure 3*a*, the ratio ${∆}_{I}/{∆}_{D}$ is plotted for different $R_{0}$ values. Three properties of this ratio are evident from equation (2.11) (and making use of the fact that Z=0 when $v\;\geq \;v_{h}$ for any specified $R_{0}$ ):

— when $v\;\geq \;v_{h}$ , the ratio ${∆}_{I}/{∆}_{D}$ can be determined by

(2.12) $$\frac{\Delta_{I}}{\Delta_{D}}\;=\;\frac{\left(1-v\right)Z^{*}-Z}{v\;Z^{*}}\;=\;\frac{1-v}{v};$$

— when ${v\geq v}_{h}$ , the ratio ${∆}_{I}/{∆}_{D}$ reaches its maximum at $v=v_{h}$ , giving

(2.13) $$\frac{\Delta_{I}}{\Delta_{D}}{|}_{\left(v\;=\;v_{h}\right)}\;=\;\frac{1-v_{h}}{v_{h}}\;=\;\frac{1}{R_{0}-1};\;\;$$

— and the ratio reaches zero when the entire population is vaccinated,

(2.14) $$\frac{\Delta_{I}}{\Delta_{D}}{|}_{\left(v\;=\;1\right)}\;=\;0.$$

In electronic supplementary material, note 3, it is shown that the indirect to direct effect ratio, when only a few individuals are vaccinated (i.e. $v\;=\;0^{+}$ ), can be calculated analytically as


(2.15) $$\frac{\Delta_{I}}{\Delta_{D}}{|}_{\left(v\;=\;0^{+}\right)}\;=\;\frac{1}{1-R_{0}\left(1-Z^{*}\right)}\;-\;1.$$

Recall that from equation (2.10), as long as $v\;\leq \;v_{h}$ , then $\Delta_{I}≃v\;Z^{*}/\left(R_{0}\;-\;1\right)$ . Thus, a simple approximation for the ratio ${∆}_{I}/{∆}_{D}$ in the outbreak phase can be obtained by


(2.16) $$\frac{\Delta_{I}}{\Delta_{D}}≃\frac{v\;Z^{*}}{\left(R_{0}-1\right)\;\left(v\;Z^{*}\right)}\;=\;\frac{1}{R_{0}-1}.$$

Figure 3*b* shows the ratio ${∆}_{I}/{∆}_{D}$ as a function of the basic reproduction number $R_{0}$ , where $R_{0}\;>\;1/\left(1\;-\;v\right)$ , for the vaccination levels v=0.1, 0.2, 0.3, 0.4 . The black points in figure 3*b* identify where v = 1 – 1/ $R_{0}$ , indicating the respective lowest $R_{0}$ required to initiate an epidemic given a vaccination level. The approximation stated by equation (2.16) is confirmed in figure 3*b* where the exact ratio ${∆}_{I}/{∆}_{D}$ is compared with the r.h.s of equation (2.16) (dotted grey curve), both versus $R_{0}$ .

Equation (2.16) shows that the indirect and direct effects of vaccination are approximately equal when $R_{0}\;=\;2$ . In the regime of $1\;<\;R_{0}\;<\;2$ , the indirect vaccination effect can be significantly larger than the direct vaccination effect. For $R_{0}\;>\;2$ , the indirect vaccination effect is always less than the direct vaccination effect, and it is almost negligible for $R_{0}\;>\;4$ . See the dotted grey line showing the relationship between the ratio and $R_{0}$ as predicted by equation (2.16).

In a related study, Eichner *et al*. [4] also examine the ratio of indirect to direct vaccination effects in an SIR model having a vaccination compartment. However, their analysis assumes the presence of demographic birth and death processes, and is carried out when the system reaches an endemic equilibrium state, rather than over a dynamic epidemic as done here. Nevertheless, and intriguingly, they still find that the ratio is given by equation (2.16) namely $\Delta_{I}/\Delta_{D}\;=\;1/\left(R_{0}\;-\;1\right)$ . Using an approximate form of the number of infected individuals for a discrete-time SIR model and examining the mortality rate of the disease, Scutt *et al*. [11] calculate the ratio ${∆}_{I}/{∆}_{D}$ for deaths averted.

### 2.3. When does the indirect vaccination effect play a major role?

The indirect vaccination effect can be substantial, in some cases exceeding 900% of the direct vaccination effect as seen in figure 3*b*. However, here, we show that this does not imply that unvaccinated individuals gain more benefit from the vaccination campaign than vaccinated individuals. To this end, we use the theoretical analysis presented so far and an intriguing example inspired by [11].

Consider a population of N=500 000 where 50 000 susceptible individuals are vaccinated ( v=0.1 ). A virus with a basic reproduction number $R_{0}\;=\;1.2$ invades the population. By solving equation (2.4) we obtain that in the ensuing epidemic, N⋅Z(0.1)=65 000 individuals become infected. Had there been no vaccine, from equation (2.5) we find the estimate indicating that $NZ^{*}\;=\;NZ\left(0\right)\;=\;157\;000$ would have been infected altogether. Thus, because of the vaccine $N\left(Z^{*}\;-\;Z\left(v\right)\right)\;=\;92\;000$ people avoided infection. This can be broken down into

— direct vaccination effect, i.e. $N\Delta_{D}\;=\;v\;N\;Z^{*}\;=\;15\;700$ infections were averted in the vaccinated population, as found from equation (2.8) and
— indirect vaccination effect, i.e. $N\Delta_{I}\;=\;76\;300$ infections were averted in the unvaccinated population, using equation (2.9).

The ratio of indirect to direct vaccination effects is thus $\left(N\Delta_{I}\right)/\left(N\Delta_{D}\right)\;=\;4.9$ . Also, equation (2.16) gives an excellent approximation $\Delta_{I}/\Delta_{D}≃1/\left(R_{0}\;-\;1\right)\;=\;5$ without any of the above calculations.

The above example is intriguing because it demonstrates that, at the population level, the indirect vaccination effect can have a significant impact and, in this case, averts a relatively large number of infections, here some five times that of the direct vaccination effect. But the ratio is slightly misleading in that the unvaccinated pool where the indirect effects take place is particularly large (450 000), in fact, nine times larger than the relatively small vaccinated pool (50 000). To clearly understand this phenomenon, it is essential to examine the effects on a ‘per capita’ basis:

— Direct vaccination effects are $\left(N\Delta_{D}\right)/\left(Nv\right)\;=\;15\;700/\;50\;000\;=\;0.314$ infections averted per vaccinated individual.
— Indirect vaccination effects are $\left(N\Delta_{I}\right)/\left[N\left(1\;-\;v\right)\right]\;=\;76\;300\;/\;450\;000\;=\;0.169$ infections averted per unvaccinated individual.

The ratio of the total vaccination effect in the unvaccinated group per unvaccinated individual, against the total vaccination effect in the vaccinated group per vaccinated individual, is the per capita ratio (PCR),


(2.17) $$PCR\;=\;\frac{N\;\Delta_{I}}{\left[\left(1\;-\;v\right)\;N\right]}/\frac{N\;\Delta_{D}}{v\;N}\;=\;\frac{v}{\left(1\;-\;v\right)}\;\frac{\Delta_{I}}{\;\Delta_{D}}.$$

Thus, even though the indirect vaccination effect can be very large compared with direct vaccination effect (here five times larger), the per capita ratio of indirect to direct vaccination effect is PCR=0.54 . This measurement clearly shows that a vaccinated individual will still benefit much more than an unvaccinated individual from vaccination, as might be expected.

### 2.4. The ‘per capita’ effect

In summary, the PCR of the total vaccination effect in the unvaccinated group to the total vaccination effect in the vaccinated group is defined as in equation (2.17), which combined with equation (2.11) gives


(2.18) $$PCR\;=\;\frac{v}{1\;-\;v}\;\frac{\Delta_{I}}{\Delta_{D}}\;=\;\frac{v}{1\;-\;v}\;\left(\frac{1\;-\;v}{v}\;-\;\frac{Z}{v\;Z^{*}}\right)\;\leq \;1.$$

Thus, the PCR is completely determined by the vaccination level v and the final size of the epidemic in the population both with and without the vaccine, i.e. Z and $Z^{*}$ , respectively.

Figure 3*c* depicts the PCR as a function of $R_{0}$ for a range of vaccination levels. It is important to note that, for a vaccine with 100% efficacy, all infections occur exclusively in unvaccinated individuals, so that indirect vaccination effects only impact the unvaccinated population. Thus, the total vaccination effect in the unvaccinated group is the indirect vaccination effect, while the total vaccination effect in the vaccinated group is the direct vaccination effect.

As seen in figure 3*c*, the PCR is always between 0 and 1. (The PCR equals unity when the vaccination level equals or exceeds the herd immunity threshold $v_{h}$ , and below the herd immunity threshold, the PCR is always smaller than 1.) Thus, the PCR indicates that the protection for unvaccinated individuals through the indirect vaccination effect cannot be larger than the direct vaccination effect for vaccinated individuals on a per capita basis. However, comparing this with the data from figure 3*b*, in the scenario where 1 < $R_{0}$ < 2, we observe that ${∆}_{I}/{∆}_{D}$ > 1, implying that the indirect vaccination effect consistently dominates the direct vaccination effect at the population level. As a result, solely focusing on the ratio ${∆}_{I}/{∆}_{D}$ can potentially exaggerate the perceived role of indirect vaccination effects, especially when the unvaccinated population is very large. Furthermore, it can be seen in figure 3*c* that given a particular $R_{0}$ , a higher v corresponds to a higher PCR, suggesting that unvaccinated individuals can benefit more from a higher vaccination coverage. In contrast, as can be seen in figure 3*b* or deduced from the approximation given in equation (2.16), the indirect to direct effect ratio ${∆}_{I}/{∆}_{D}$ is almost independent of the vaccination level. This clearly shows how ${∆}_{I}/{∆}_{D}$ can lead to misconceptions and highlights the importance of PCR for the analysis of the effects of vaccination.

## 3. Shielding model of epidemics

In 2020, the SARS-CoV-2 virus emerged and led to a devastating global pandemic over the next years. Initially, and for at least a year, no vaccination was available for protection—it appeared the entire world population was susceptible. In the absence of vaccination, Weitz *et al*. [5] devised a mitigation strategy based on the fact that recovered individuals will always gain (at least short-term) immunity and can be used as ‘shields’ to limit SARS-CoV-2 transmission. Here, we will examine different shielding strategies and discuss the role of indirect effects.

### 3.1. Shielding through recovered individuals

In practice, the strategy attempts to place susceptible members of the population as close as possible to anyone who has recovered from the disease. In other words, a susceptible individual will then tend to come into contact with recovered individuals more than other susceptible or infected individuals. In theory, the recovered individual will act to block transmission and reduce the number of chains of infection that could potentially reach the susceptible. The epidemic final size would also be expected to reduce. By relocating individuals and their contacts, the population mixing becomes non-random and thus more difficult to model. Nevertheless, Weitz *et al*. [5] devised a simple model to approximate the ‘shielding’ effect. Their model assumes that there is a relative preference of 1+α that a given individual will interact with a recovered individual in what would be otherwise an interaction with a random individual. This type of interaction substitution is equivalent to assuming an effective contact rate ratio of 1+α for recovered individuals relative to the rest of the population. The larger the α , the more the chance that a susceptible individual is in the vicinity of a recovered individual, and therefore the larger the shielding effect.

The following modified SIR equations [5] involve shielding into the:


(3.1) $$\begin{array}{l} \frac{dS}{dt}\;=\;-\beta \frac{SI}{1\;+\;\alpha R},\\ \frac{dI}{dt}\;=\;\beta \frac{SI}{1\;+\;\alpha R}\;-\;\gamma I,\\ \frac{dR}{dt}\;=\;\gamma I, \end{array}$$

where S , I , R are the fractions of susceptible, infectious and recovered individuals, respectively, and S(0)=1 , I(0)=R(0)=0 . Given that S+I+R=1 , the term 1+αR can be thought of as S+I+(1+α)R . As described in [5], this assumes an effective contact ratio of 1+α for a recovered individual relative to the rest of the population ( S+I ). The transmission rate is given by the term β/[1+αR(t)] , and thereby clearly, the more recovered individuals there are, or the higher the α , the lower will be the epidemic transmission through the population. The time-varying basic reproduction number is defined as $R_{0}\left(t\right)\;=\;\beta /\left[\left(1\;+\;\alpha R\left(t\right)\right)\cdot \gamma \right]$ , where β is the infection rate and γ is the recovery rate.

**Figure 4.**
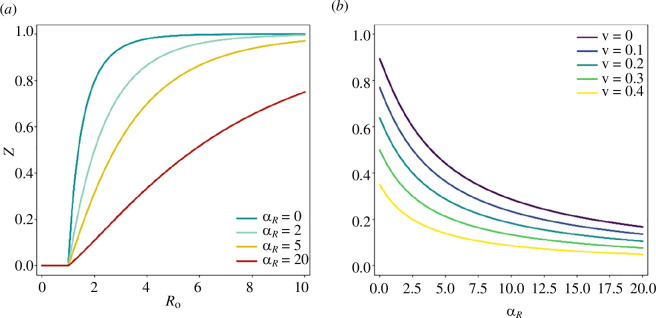
(*a*) The final size of the epidemic Z as a function of the basic reproduction number $R_{0}$ for initial vaccination coverage v=0 with shielding strength $\alpha \;=\;\alpha_{R}\;=\;0,\;2,\;5,\;20$ . (*b*) The final size of the epidemic Z as a function of the shielding strength α (or $\alpha_{R}$ , since shielding is implemented on recovered individuals only) for $R_{0}\;=\;2.5$ , separately for different vaccination levels v , calculated by solving equations (3.4) and (3.5).

Through numerical simulations, Weitz *et al*. [5] show that shielding acts to reduce the epidemic peak and shortens the duration of the epidemic spread. By directly solving the following equations (3.4) and (3.5), without relying on numerical simulations, we show the effect of shielding on the final size of the epidemic as a function of the basic reproduction number $R_{0}\;=\;\beta /\gamma$ in figure 4*a*, and as a function of shielding strength α in figure 4*b*. It is clear from the results that very large levels of shielding are required to make a substantial impact on the size of the epidemic. An important observation is that any shielding, no matter how strong (even for α=20 ), slows down the epidemic but never stops it completely (i.e. by crossing the herd immunity threshold). Intuitively, this might be expected, since in situations when there are few recovered individuals available to provide protection, shielding becomes less effective. In other words, shielding via ‘recovered’ individuals depends both on shielding strength and the number of recovered individuals. However, as shielding strength increases and slows down the epidemic, fewer individuals are infected and thus there are fewer recovered individuals, which in turn decreases the effectiveness of shielding. This is illustrated in figure 4*b*, where increasing the shielding strength eventually loses its effect asymptotically.

### 3.2. Shielding through both vaccinated and recovered individuals

Here we extend the shielding strategy [5] to a more general mitigation strategy where some (even limited) vaccination is available and thus both recovered and/or vaccinated individuals can be used as shields. In the *extended shielding model*, a susceptible individual has a relative preference of $1\;+\;\alpha_{V}$ for contact with a vaccinated and $1\;+\;\alpha_{R}$ with a recovered individual relative to other individuals. The new model can be formally presented with the following equations:


(3.2) $$\begin{array}{ll} \frac{dS}{dt}\; & =\;-\beta \frac{SI}{1\;+\;\alpha_{V}v\;+\;\alpha_{R}R},\\ \frac{dI}{dt}\; & =\;\beta \frac{SI}{1\;+\;\alpha_{V}v\;+\;\alpha_{R}R}\;-\;\gamma I,\\ \frac{dR}{dt}\; & =\;\gamma I,\\ \frac{dN}{dt}\; & =\;\alpha_{R}\gamma I. \end{array}$$

The function $N\left(t\right)\;=\;1\;+\;\alpha_{V}v\;+\;\alpha_{R}R\left(t\right)$ plays a key role in the analysis of the model. The fraction of vaccinated individuals is set as a constant v , and thus S(0)=1−v , I(0)=R(0)=0 . Similar to the base shielding model (equation (3.1)), given S+I+R+v=1 , the term $1\;+\;\alpha_{V}v\;+\;\alpha_{R}R$ can be thought of as $S\;+\;I\;+\;\left(1+\alpha_{V}\right)v\;+\;\left(1+\alpha_{R}\right)R$ . Then, the transmission rate is given by the term $\beta /\left[1\;+\;\alpha_{V}v\;+\;\alpha_{R}R\left(t\right)\right]$ , and thus, the time-varying basic reproduction number is given by $R_{0}\left(t\right)\;=\;\beta /\left[\left(1\;+\;\alpha_{V}v\;+\;\alpha_{R}R\left(t\right)\right)\gamma \right]$ , where β is the infection rate and γ is the recovery rate.

### 3.3. Analysis of the extended shielding model

The extended shielding model (equation (3.2)) can be investigated analytically to characterize the effect of shielding when both vaccinated and recovered populations are participating. Here, we examine the relationship between the final size of the epidemic ( Z ) and the shielding strength $\alpha_{V}$ and $\alpha_{R}$ .

#### 3.3.1. Shielding via vaccinees only ( $\alpha_{V}\;>\;0,\;\;\alpha_{R}\;=\;0$ )

This is the case where recovered individuals are not used as shields and the shielding is based entirely on vaccinated individuals. As before, we assume that at t=0 , initial conditions are S(0)=1−v , I(0)=R(0)=0 . Since $\alpha_{R}\;=\;0$ , the system in equation (3.2) is equivalent to a standard SIR epidemic except now with $R_{0}\left(v\right)\;=\;\beta /\left[\left(1\;+\;\alpha_{V}v\right)\gamma \right]$ . Thus, the effective reproduction number is given by $R_{e}\left(t\right)\;=\;R_{0}\left(v\right)\cdot S\left(t\right)$ , and an epidemic will occur at t=0 only if $R_{e}\left(0\right)\;>\;1$ , so that $I\left(t\right)$ would have initial positive growth. The condition for epidemic breakout can be worked out for the vaccinated-shielding strength as


(3.3) $$\alpha_{V}\;<\;\left[\beta \left(1\;-\;v\right)/\gamma \;-\;1\right]/v.$$

The final size of the epidemic $Z\left(v\right)$ is as before defined by equation (2.4). Thus, increasing vaccinated-shielding strength ( $\alpha_{V}$ ) decreases $R_{0}$ , and this in turn decreases the final size Z until it reaches zero when $\alpha_{V}\;=\;\left[\beta \left(1\;-\;v\right)/\gamma \;-\;1\right]/v$ . This means that with a large enough vaccinated shielding, an epidemic can be prevented.

#### 3.3.2. Shielding via both vaccinees and recovered individuals ( $\alpha_{V},\;\alpha_{R}\;>\;0$ )

The equilibrium of equation (3.2) occurs when all time derivatives are set to 0. Note that at equilibrium we must have $\lim_{t\to \infty }I\left(t\right)\;=\;I^{*}\;=\;0$ . Suppose also that at equilibrium $\lim_{t\to \infty }N\left(t\right)\;=\;N^{*}$ and $\lim_{t\to \infty }R\left(t\right)\;=\;R^{*}$ . Since the final size of the epidemic Z is exactly $R^{*}$ , and $N^{*}\;=\;1\;+\;\alpha_{V}v\;+\;\alpha_{R}R^{*}$ , thus Z can be derived as


(3.4) $$Z\;=\;\;\left(N^{*}\;-\;1\;-\;\alpha_{V}v\right)/\alpha_{R}.$$

Our analysis of the extended shielding model (equation (3.2)) in electronic supplementary material, note 4, reveals that $N^{*}$ satisfies the equation


(3.5) $$N^{*c+1}\;-\;\left(\alpha_{R}\left(1-v\right)\;+\;1\;+\;\alpha_{V}v\right)N^{*c}\;+\;\alpha_{R}\left(1-v\right)\;{\left(1\;+\;\alpha_{V}v\right)}^{c}\;=\;0,$$

where $c\;=\;\beta /\left(\alpha_{R}\gamma \right)$ and $1\;+\;\alpha_{V}v\;\leq \;N^{*}\;\leq \;1\;+\;\alpha_{V}v\;+\;\alpha_{R}\left(1-v\right)$ . In electronic supplementary material, note 4, we show in detail that there is always only one root of equation (3.5) that satisfies the range of $N^{*}$ . Using numerical procedures, equation (3.5) can be solved to obtain the unique value of $N^{*}$ , and substituting $N^{*}$ in equation (3.4) gives the final size of the epidemic Z . For this scenario, the time-varying basic reproduction number is given by $R_{0}\left(t\right)\;=\;\beta /\left[\left(1\;+\;\alpha_{V}v\;+\;\alpha_{R}R\left(t\right)\right)\gamma \right]$ , the effective reproduction number is given by $R_{e}\left(t\right)\;=\;R_{0}\left(t\right)\;S\left(t\right)$ , and an epidemic will occur only if $R_{e}\left(t\right)\;>\;1$ at t=0 , i.e. $\alpha_{V}\;<\;\left[\beta \left(1\;-\;v\right)/\gamma \;-\;1\right]/v$ . (Note that the condition for epidemic breakout is the same as equation (3.3) calculated for the case where $\alpha_{R}\;=\;0$ , investigated earlier.)

**Figure 5.**
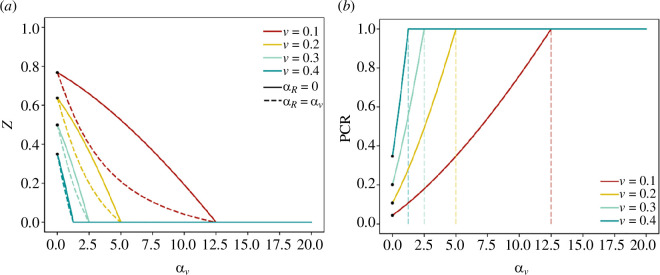
(*a*) The final size of the epidemic Z as a function of the shielding strength $\alpha_{V}$ for different vaccination levels v=0, 0.1, 0.2, 0.3, 0.4 , separately demonstrated for no recovered shielding $\alpha_{R}\;=\;0$ (solid curves), and increasing recovered shielding $\alpha_{R}\;=\;\alpha_{V}$ (dashed curves). (*b*) PCR as a function of vaccinated-shielding strength $\alpha_{V}$ for different vaccination levels v=0, 0.1, 0.2, 0.3, 0.4 . The results in both panels are calculated based on the PCR definition in equation (2.18) and the solution of equations (3.4) and (3.5); all data are generated by setting $R_{0}\;=\;2.5$ .

The effect of shielding mediated via vaccinated individuals is demonstrated in the results seen in figure 5*a*, obtained by applying equations (3.4) and (3.5) derived for the extended shielding model formulation (equation (3.2)). The solid curves represent the case where there is no recovered shielding ( $\alpha_{R}\;=\;0$ ) and dashed curves represent the case where recovered- and vaccinated-shielding strengths increase together ( $\alpha_{R}\;=\;\alpha_{V}$ ). As one can expect, the larger the $\alpha_{V}$ , the smaller the final size of the epidemic. Clearly, for different vaccination levels colour-coded in figure 5*a*, the final size of the epidemic reaches zero only when the vaccinated-shielding strength crosses the herd immunity threshold $\alpha_{V}\;=\;\left[\beta \left(1\;-\;v\right)/\gamma \;-\;1\right]/v$ (see equation (3.3)), regardless of the recovered-shielding strength $\alpha_{R}$ . Moreover, the results in figure 5*a* show that for a higher initial vaccination coverage v , the herd immunity threshold for vaccinated-shielding strength $\alpha_{V}$ is lower (consistent with equation (3.3)). In other words, the higher the vaccination level, the less the vaccinated shielding required to achieve herd immunity. Interestingly, adding recovered shielding further reduces the final size between $0\;\leq \;\alpha_{V}\;<\;\left[\beta \left(1\;-\;v\right)/\gamma \;-\;1\right]/v$ , but only by increasing $\alpha_{V}$ is it possible that $R_{e}\left(0\right)\;\leq \;1$ , which would completely curb the epidemic. This is seen by the solid and dashed curves of each particular colour (corresponding to a particular vaccination level) reaching Z=0 at the same $\alpha_{V}$ threshold independent of $\alpha_{R}$ . In electronic supplementary material, note 4, we use numerical simulations to find the relationship between the final size of the epidemic and shielding strength parameters ( $\alpha_{V}$ and $\alpha_{R}$ ) and obtain identical results that confirm those shown in figure 5*a*.

It is intuitive that using vaccinated individuals as shields can increase the indirect vaccination effect, thereby increasing the PCR. It is thus helpful to use PCR to explain the curbing effect of the vaccinated-shielding strength $\alpha_{V}$ on epidemics. In figure 5*b*, we show the effect of shielding on the PCR as a function of the shielding strength $\alpha_{V}$ , for different levels of vaccination v in the initial population. As seen in figure 5*b*, increasing vaccinated-shielding strength $\alpha_{V}$ directly increases the PCR, with higher vaccination levels strengthening this effect. Also, the results confirm the epidemic breakout (or herd immunity if viewed inversely) condition in equation (3.3), with PCR reaching unity exactly at $\alpha_{V}\;=\;\left[\beta \left(1\;-\;v\right)/\gamma \;-\;1\right]/v$ (marked by the dashed lines associated with different vaccination levels).

## 4. Imperfect vaccine

With an ideal perfect or near-perfect vaccine that is 100% effective, vaccinated individuals will (almost) never become infected. However, vaccines are often imperfect lacking efficacy in different ways, and we allow for that in our analysis here. Suppose that initially a proportion v ( 0<v≤1 ) of a fully susceptible population has been vaccinated with an imperfect vaccine with an efficacy *k* (0 < *k* < 1). The vaccine’s imperfect efficacy will be modelled in two different ways, namely, all-or-nothing and leaky vaccines. Additionally, we examine the impact of vaccine efficacy on the final size of the epidemic and the final size of severe disease infections (see electronic supplementary material, note 6 for details).

### 4.1. All-or-nothing vaccines

‘All-or-nothing’ vaccines, provide a fraction *k* of the vaccinated individuals with full immunity and the remaining fraction 1 − k, remain completely susceptible to infection [17,18]. This is equivalent to assuming that a fraction kv of a fully susceptible population is initially vaccinated and thus fully protected from infection. Consequently, many properties can be derived directly from the SIR model for a perfect vaccine but swapping vaccine coverage to kv rather than v . See table 1 for different properties of perfect and all-or-nothing vaccines that are derived by this swap. For all-or-nothing vaccines, we separately calculate the total vaccination effect in the vaccinated ( ${∆}_{TV}$ ) and unvaccinated ( ${∆}_{TU}$ ) population. The total vaccination effect in the vaccinated group ( ${∆}_{TV}$ ) includes both the direct vaccination effect ( ${∆}_{D}$ ), which occurs only in those who were effectively vaccinated, and a fraction of the indirect vaccination effect ( ${∆}_{I}$ ), which occurs in those who were ineffectively vaccinated. Thus $\Delta_{TV}\;=\;\Delta_{D}\;+\;\frac{\left(1\;-\;k\right)v}{1\;-\;v\;+\;\left(1\;-\;k\right)v}\Delta_{I}$ , where $\Delta_{D}\;=\;kv\;Z^{*}$ and $\Delta_{I}\;=\;Z^{*}\;-\;Z\left(kv\right)\;-\;kv\;Z^{*}$ . The total vaccination effect in the unvaccinated group ( ${∆}_{TU}$ ), which is the fraction of the indirect vaccination effect that occurs in the unvaccinated population, is $\Delta_{TU}\;=\;\frac{1\;-\;v}{1\;-\;v\;+\;\left(1\;-\;k\right)v}\;\Delta_{I}$ . This is because the indirect vaccination effect is distributed equally among unvaccinated and the ineffectively vaccinated. These effects, ${∆}_{TV}$ and ${∆}_{TU}$ , are used to calculate the PCR (see table 1) based on its definition in §2.4.

**Table 1.** Different properties of perfect and imperfect vaccines derived from mathematical analysis. See electronic supplementary material, note 5 for the derivation of herd immunity threshold ( $v_{h}$ ), total vaccination effect ( ${∆}_{T}$ ), direct vaccination effect (Δ_D_), indirect vaccination effect ( ${∆}_{I}$ ) and per capita ratio (PCR).

vaccine type	$v_{h}$	$\Delta_{T}$	$\Delta_{D}$	$\Delta_{I}$	PCR
perfect vaccine	$v_{h}\;=\;1\;-\;\frac{1}{R_{0}}$	$Z^{*}\;-\;Z\left(v\right)$	${vZ}^{*}$	$Z^{*}\;-\;Z\left(v\right)\;-\;v\;Z^{*}$	$\frac{v}{1\;-\;v}\cdot \frac{\Delta_{I}}{\Delta_{D}}$
‘all-or-nothing’ vaccine	if $k\;\geq \;1\;-\;\frac{1}{R_{0}}$ , $v_{h}\;=\;\frac{1\;-\;\frac{1}{R_{0}}}{k}$ ; otherwise, none.	$Z^{*}\;-\;Z\left(kv\right)$	${kvZ}^{*}$	$Z^{*}\;-\;Z\left(kv\right)\;-\;kv\;Z^{*}$	$\frac{v}{\left(1\;-\;v\right)}\cdot \frac{\Delta_{TU}}{\Delta_{TV}}$
‘leaky’ vaccine	if $k\;\geq \;1\;-\;\frac{1}{R_{0}}$ , $v_{h}\;=\;\frac{1\;-\;\frac{1}{R_{0}}}{k}$ ; otherwise, none.	$Z^{*}\;-\;Z\left(v\right)$	$v\left(e^{-\left(1\;-\;k\right)R_{0}\;Z^{*}}\;-\;e^{-R_{0}\;Z^{*}}\right)$	$Z^{*}\;-\;Z\left(v\right)\;-\;v\left(e^{-\left(1-k\right)R_{0}\;Z^{*}}\;-\;e^{-R_{0}\;Z^{*}}\right)$	$\frac{v}{\left(1\;-\;v\right)}\cdot \frac{\Delta_{TU}}{\Delta_{TV}}$

### 4.2. Leaky vaccines

Leaky vaccines provide partial protection to each vaccinated individual, resulting in a reduced relative susceptibility (1 − *k*) compared with unvaccinated individuals [17,18]. Similar to Shim & Galvani [18], an S_U_S_V_I_U_I_V_R_U_R_V_ model is used here to model leaky vaccines. Table 1 summarizes different properties for the model with leaky vaccines, with all the details provided in electronic supplementary material, note 5. For leaky vaccines, we find that (see electronic supplementary material, note 5) the final size of the epidemic satisfies $Z\left(v\right)\;=\;\left(1\;-\;v\right)\left[1\;-\;e^{-R_{0}\;Z\left(v\right)}\right]\;+\;v\left[1\;-\;e^{-\left(1-k\right)R_{0}\;Z\left(v\right)}\right]$ , the total vaccination effect in the unvaccinated group is $\Delta_{TU}\;=\;\left(1\;-\;v\right)Z^{*}\;-\;\left(1\;-\;v\right)\left[1\;-\;e^{-R_{0}\;Z\left(v\right)}\right]$ and the total vaccination effect in the vaccinated group is $\Delta_{TV}\;=\;\Delta_{T}\;-\;\Delta_{TU}$ .

For a perfect vaccine (see §3.3), if $v\;<\;v_{h}\;=\;1\;-\;\frac{1}{R_{0}}$ , increasing the vaccinated-shielding strength ( $\alpha_{V}$ ) to $\alpha_{V}\;=\;\frac{\left(1\;-\;v\right)R_{0}\;-\;1}{v}$ can prevent an epidemic. Similarly, for ‘all-or-nothing’ vaccines, if $kv\;<\;1\;-\;\frac{1}{R_{0}}$ , increasing the shielding strength $\alpha_{V}$ to $\alpha_{V}\;=\;\frac{\left(1\;-\;kv\right)R_{0}\;-\;1}{kv}$ can prevent an epidemic. However, shielding via leaky vaccines may not always reduce the final size (*Z*) of the epidemic (as shown through numerical simulations of the shielding model with ‘leaky’ vaccinees in electronic supplementary material, note 5). Whether an epidemic can be prevented by shielding via ‘leaky’ vaccinees, depends not only on the shielding strength $\alpha_{V}$ but also on the vaccine efficacy k and the vaccination level v . As worked out in electronic supplementary material, note 5, if $kv\;\geq \;1\;-\;1/R_{0}$ , the final size of the epidemic is *Z* = 0. In the case $kv\;<\;1\;-\;\frac{1}{R_{0}}$ , we can show the following: if both $k\;>\;1\;-\;\frac{1}{R_{0}}$ and $\alpha_{V}\;\geq \;\frac{R_{0}\left(1\;-\;kv\right)\;-\;1}{v\left[1\;-\;R_{0}\left(1\;-\;k\right)\right]}$ , then the final size of the epidemic is *Z* = 0, and *Z* > 0 otherwise. Thus, to prevent an epidemic, or in other words, to achieve a final size (*Z*) of 0 by increasing the shielding strength of leaky vaccinees, the efficacy of leaky vaccines k must be large enough such that $k\;>\;\left(1\;-\;\frac{1}{R_{0}}\right)$ .

### 4.3. Per capita ratio for imperfect vaccines

**Figure 6.**
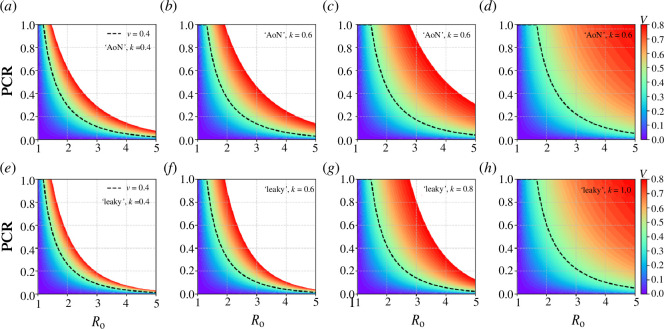
The PCR plotted as a function of $R_{0}$ for different vaccination levels v colour coded between 0 and 0.8, and different vaccine efficacies *k* of 0.4, 0.6, 0.8 and 1. This is plotted for both ‘all-or-nothing’ (AON) (*a–d*) and ‘leaky’ (*e–h*) vaccines. The dashed black curve shows the trend in PCR versus increasing $R_{0}$ for v=0.4 .

Recall that the PCR is the total vaccination effect in the unvaccinated group per unvaccinated individual against the total vaccination effect in the vaccinated group per vaccinated individual. Using the formulae given in §§4.1 and 4.2 for ‘all-or-nothing’ and ‘leaky’ vaccines, we demonstrate how the efficacy *k* of these vaccines affects the PCR. In figure 6, the PCR is plotted as a function of $R_{0}$ for different vaccination levels v (colour coded between 0 and 0.8) and different vaccine efficacies *k* (*k* = 0.4, 0.6, 0.8 and 1) for both ‘all-or-nothing’ and ‘leaky’ vaccines. Note that when the efficacy of vaccines is set to *k* = 1 represents a perfect vaccine. By comparing figure 6*a–h*, we observe that for the same vaccine efficacy and vaccination level, the PCR is higher for the ‘all-or-nothing’ type than for the ‘leaky’ type. Additionally, regardless of whether it is an ‘all-or-nothing’ or ‘leaky’ vaccine, higher vaccine efficacy and higher vaccination level always lead to a higher PCR, indicating that each unvaccinated individual will benefit more from vaccination. However, the PCR is always less than or equal to 1, indicating that no matter the vaccine efficacy (in both all-or-nothing and leaky types) a vaccinated individual will always benefit more from vaccination than an unvaccinated individual.

## 5. Discussion and conclusion

Unlike the approaches used in previous studies, such as the equilibrium analysis of Eichner *et al*. [4] and the approximate discrete-time SIR model of Scutt *et al*. [11], this article adopts the final size formula to explore the impact of vaccination. This method makes it possible to obtain many useful results analytically and unpack vaccination effects and mitigation strategies. Our results indicate that the ratio of indirect to direct vaccination peaks at the herd immunity threshold $v_{h}\;=\;1\;-\;\frac{1}{R_{0}}$ (figure 3*a*), with the ratio being substantial for outbreaks with a lower vaccination coverage v (figure 3*a*,*b*). Although the indirect vaccination effect can be substantial—potentially many times greater than the direct vaccination effect—the presence of a large unvaccinated population can change how this should be interpreted. The influence of the size of the unvaccinated population is reflected better in the PCR, i.e. the per capita ratio of the total vaccination effect in the unvaccinated group to the total vaccination effect in the vaccinated group. As expected, the PCR measure reveals that the benefits of the total vaccination effect for an unvaccinated individual can never surpass the total protection of the vaccine for a vaccinated individual.

Either limiting the transmission of the infectious disease or reducing the number of susceptible individuals (e.g. through vaccination) can effectively reduce the final size of the epidemic. While the strategy of using recovered individuals as shields to limit the transmission of SARS-CoV-2 has been widely discussed [19–21], it has the disadvantage of requiring large-scale serologic testing, making it almost impractical [5]. With regard to vaccination, achieving an initial coverage beyond the herd immunity threshold can reduce the final size of the epidemic to zero, but that usually requires vaccinating a substantial proportion of a population. Moreover, vaccine availability may be limited, especially in less developed countries. Our proposed extension of the shielding model can be an effective response. It suggests using vaccinated individuals as shields without the help of, or in addition to, the shielding of the recovered population. This model achieves the goal of reducing the final size of the epidemic to zero for any non-zero vaccination coverage, given that the shielding strength via the vaccinated population $\alpha_{V}$ is sufficiently large, i.e. $\alpha_{V}\;\geq \;\frac{\left(1\;-\;v\right)R_{0}\;-\;1}{v}$ . Tracking vaccinated individuals is more feasible than tracking recovered individuals, making the extended shielding a more practical mitigation strategy, assuming a vaccine is available.

Our analysis covers the more realistic imperfect vaccine types, including ‘all-or-nothing’ [18,22,23] or ‘leaky’ [18,22,23]. For ‘all-or-nothing’ vaccines, we find that many results can be derived directly from the SIR model for a perfect vaccine by assuming a vaccine coverage lowered by the efficacy k of the imperfect vaccine. Through mathematical analysis, we also derived expressions for the total vaccination effect, direct vaccination effect, indirect vaccination effect and PCR for ‘leaky’ vaccines.

Additionally, we demonstrate the conditions that the shielding strength ( $\alpha_{V}$ ), vaccine efficacy ( k ) and vaccination level ( v ) must satisfy to prevent an epidemic by shielding via ‘leaky’ vaccinees. Numerical simulations (see electronic supplementary material, note 5 for details) revealed that shielding via ‘leaky’ vaccinees does not always decrease the final size *Z* of the epidemic; this depends on the shielding strength ( $\alpha_{V}$ ), vaccine efficacy ( k ) and vaccination level ( v ). Kraay *et al*. [24] found that shielding via imperfect vaccinees might not necessarily result in fewer deaths. This is because an imperfect vaccine can create ‘false positives’, where individuals who are vaccinated but not fully protected return to pre-pandemic levels of social interaction, thereby increasing their risk of infection and transmission. We also extend our method to examine the effect of the vaccine on the final size of severe disease infections (see electronic supplementary material, note 6 for details). It is found that, compared with a vaccine that can completely block transmission, a vaccine that fails to block transmission but completely prevents severe disease still leads to a larger final size (of severe infections). This is because, with the second vaccine, vaccinated individuals can still transmit the disease, and thus vaccination cannot provide indirect protection to unvaccinated individuals, leaving them at a higher risk of severe disease.

It is important to mention that our study primarily focused on epidemics with fixed $R_{0}$ and initial vaccination coverage v , assuming a 100% vaccination efficacy. In more realistic scenarios, these parameters change over time instead of remaining constant. Therefore, in certain extreme cases, our main conclusions regarding indirect and direct vaccination effects may not be applicable. For instance, if no one is vaccinated before the epidemic and the rate of vaccination during the epidemic is slow, the epidemic may conclude before a significant fraction of the population is vaccinated. Additionally, the rate of immunity loss from infection or vaccination will also impact the results [9,25,26]. These complications suggest important future research directions.

## Data Availability

All data and models are provided in the manuscript and electronic supplementary material [27].
